# Securing the surgical field for mobilization of right-sided colon cancer using the duodenum-first multidirectional approach in laparoscopic surgery

**DOI:** 10.1007/s10151-021-02444-5

**Published:** 2021-05-13

**Authors:** K. Nagayoshi, S. Nagai, K. P. Zaguirre, K. Hisano, M. Sada, Y. Mizuuchi, M. Nakamura

**Affiliations:** Department of Surgery and Oncology, Graduate School of Medical Sciences, Kyushu University, 3-1-1 Maidashi, Higashi-ku, Fukuoka, 812-8582 Japan

**Keywords:** Right colectomy, Approach, Colon cancer, Mobilization

## Abstract

**Background:**

The aim of this study was to compare the short-term outcomes of the duodenum-first multidirectional approach (DMA) in laparoscopic right colectomy with those of the conventional medial approach to assess its safety and feasibility.

**Methods:**

This retrospective study enrolled 120 patients who had laparoscopic surgery for right-sided colon cancer in our institution between April 2013 and December 2019. Fifty-four patients underwent colectomy using the multidirectional approach; among these, 20 underwent the DMA and 34 underwent the caudal-first multidirectional approach (CMA). Sixty-six patients underwent the conventional medial approach. Complications within 30 days of surgery were compared between the groups.

**Results:**

There were 54 patients in the multidirectional group [29 females, median age 72 years (range 36–91 years)] and 66 in the medial group [42 females, median age 72 years (range 41–91 years)]. Total operative time was significantly shorter in multidirectional approach patients than conventional medial approach patients (208 min vs. 271 min; *p* = 0.01) and significantly shorter in patients who underwent the DMA compared to the CMA (201 min vs. 269 min; *p* < 0.001). Operative time for the mobilization procedure was also significantly shorter in patients who underwent the DMA (131 min vs. 181 min; *p* < 0.001). Blood loss and incidence of postoperative complications did not differ. In 77 patients with advanced T3/T4 tumors, the DMA, CMA, and conventional medial approach were performed in 13, 21, and 43 patients, respectively. Total operative time and operative time of the mobilization procedure were significantly shorter in patients undergoing DMA. Blood loss and incidence of postoperative complications did not differ. R0 resection was achieved in all patients with advanced tumors.

**Conclusions:**

The DMA in laparoscopic right colectomy is safe and feasible and can achieve R0 resection with a shorter operative time than the conventional medial approach, even in patients with advanced tumors.

**Supplementary Information:**

The online version contains supplementary material available at 10.1007/s10151-021-02444-5.

## Introduction

**Fig. 1:**
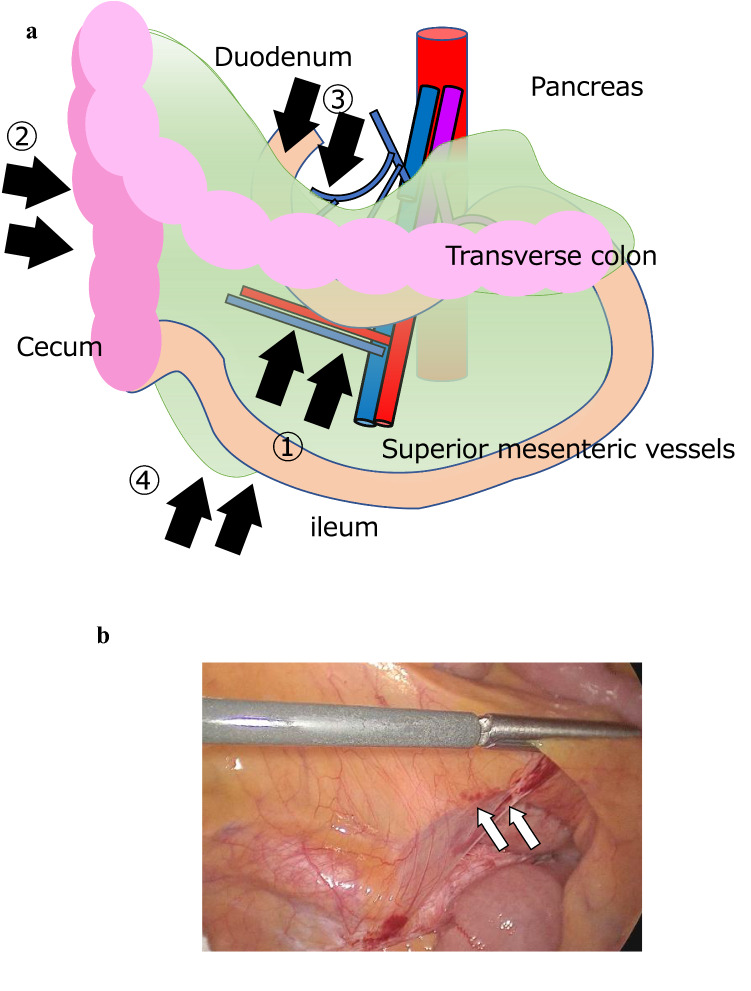
Surgical approaches for laparoscopic dissection of the right colon. **a** The schematic diagram shows the first part of each approach. The medial-to-lateral approach starts with resection of the mesentery near the ileocolic vessels (arrow 1). The lateral-to-medial approach starts from the lateral side of the cecum and the ascending colon (arrow 2). The cranial-to-caudal approach starts from the ventral side of the pancreas head (arrow 3). The retroperitoneal approach starts from the root of the mesentery of the ileum (arrow 4). **b** Mesentery dissection begins by cutting the peritoneum along the root of the mesentery above the horizontal portion of the duodenum in the duodenum-first multidirectional approach (white arrows). In the caudal-first multidirectional approach, dissection begins from the caudal side of the mesenteric root (black arrows)

Complete mesocolic excision (CME) and central vessel ligation (CVL) is a well-known concept that can reduce local recurrence and improve oncological outcomes in colon cancer surgery [1]. Previous studies have proven the feasibility and oncological and technical safety of laparoscopic right radical colectomy with CME and CVL [2, 3]. Laparoscopic operations, including right colectomy, are commonly performed for colorectal surgery in Japan [4]. Several laparoscopic right colectomy approaches have been adopted: medial-to-lateral, lateral-to-medial, cranial-to-caudal, and retroperitoneal (Fig. 1a) [5–7]. The traditional approach is medial-to-lateral [8–10], which has been standardized and achieves good oncological results comparable with the conventional lateral approach [11–13]. However, the medial-to-lateral approach requires numerous variations because of anatomical complexity around the pancreas and is considered more complex and technically difficult [14] Therefore, the superior laparoscopic approach for right-sided colon cancer remains controversial. Moreover, indications for the different approaches have not been established. A safe reproducible technique that can be widely applied by laparoscopic surgeons is needed. We have used an adapted multidirectional approach, the duodenum-first multidirectional approach (DMA) that consists of three steps and combines the advantages of the various approaches. Herein, we describe our technique and compare it with the conventional medial approach to evaluate its feasibility and safety.

## Materials and methods

This retrospective single-center study enrolled 120 patients who underwent laparoscopic surgery for right-sided colon cancer between April 2013 and December 2019 in the Department of Surgery and Oncology, Kyushu University Hospital. Sixty-six patients underwent colectomy using the conventional medial approach from 2013 to 2016, when we began performing the multidirectional technique. From 2016 to 2017, we used the caudal-first multidirectional approach (CMA) before establishing the duodenum-first multidirectional approach (DMA) technique in 2018. Therefore, a total of 54 patients underwent colectomy using the multidirectional approach: 34 patients underwent CMA and 20 underwent DMA. All operations were performed by 1 of 4 gastrointestinal surgeons with an assistant. The patients’ clinicopathological characteristics were obtained from medical records. Lymph node dissection, including the area around the root of the main feeding vessels, was performed in patients with large tumors suspected to invade beyond the deep submucosal layer, as advocated by the Japanese Guidelines for the Treatment of Colorectal Cancer [15]. Total operative time and operative time of the mobilization procedure (time from mesentery incision to completion of mobilization and CVL) were obtained from surgical videos and the anesthetic records. The number of times the assistant moved either or both hands to adjust retracting instruments to optimize the surgical field during mobilization was recorded from surgical videos. Complications within 30 days of surgery were evaluated according to the Clavien–Dindo classification [16]. This study was approved by the Kyushu University Hospital Human Research Ethics Committee (No. 29-292) and conducted in accordance with the principles of the Declaration of Helsinki.

### Mobilization using the multidirectional approach

Five ports were placed as follows: umbilical Hasson port; two 5 mm ports placed parallel in the right upper and lower quadrants; one 5 mm port placed in the left lower quadrant; and one 12 mm port placed in the left upper quadrant. Capnoperitoneum was established with intra-abdominal pressure set at 10 mm Hg. The patient was placed in the Trendelenburg position with the left side down to move the small intestines into the upper left abdomen.

**Fig. 2:**
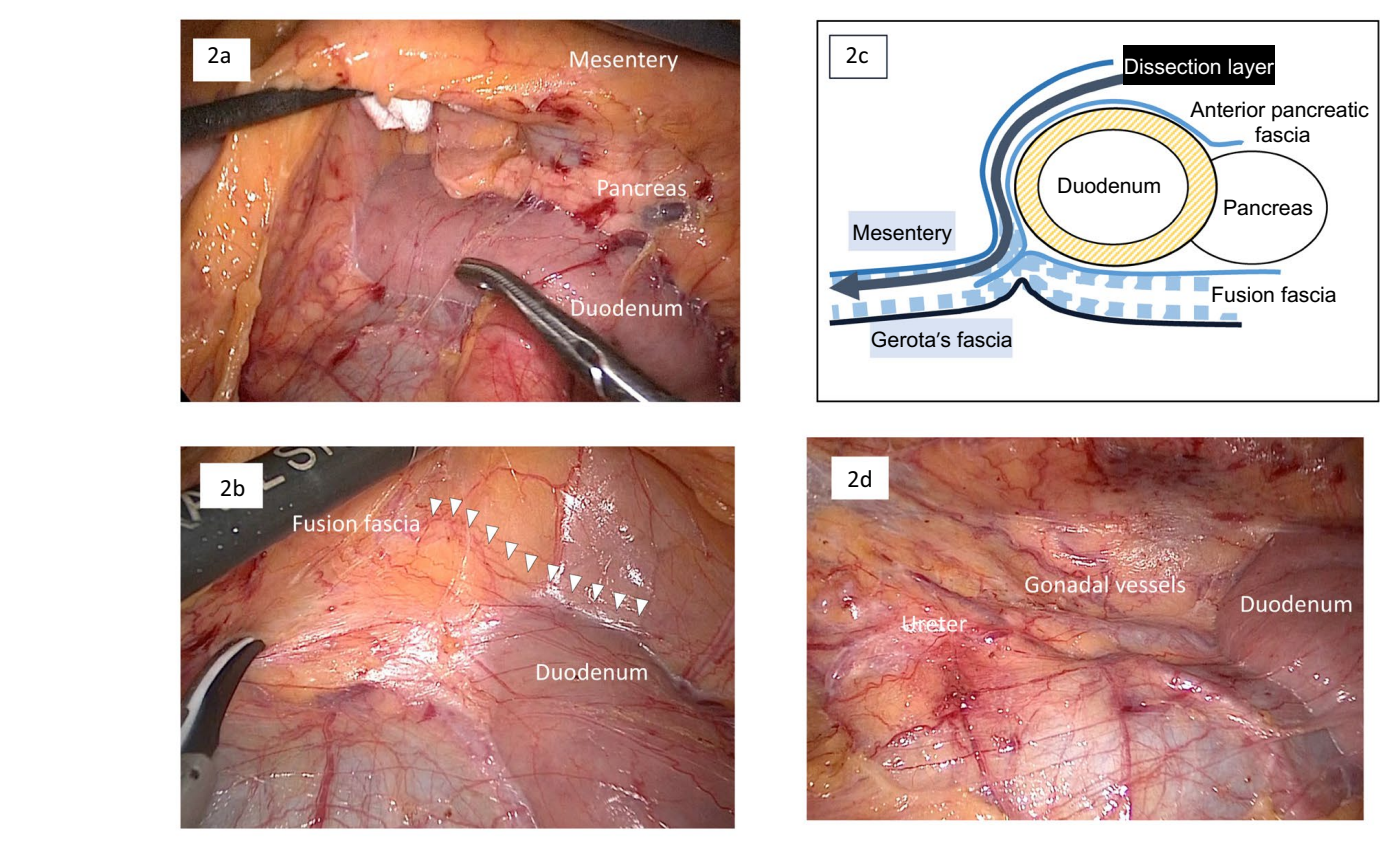
Dissection stages in the duodenum-first multidirectional approach. **a** Mesenteric dissection begins with the ventral layer of the horizontal duodenum. **b** The anterior pancreatic fascia (white arrowheads) connects with Toldt’s fusion fascia. **c** Dissection proceeds from the layer above the anterior pancreatic fascia, maintaining the layer above Gerota’s fascia. **d** Full view after dorsal dissection, with retroperitoneal organs preserved

**Fig. 3:**
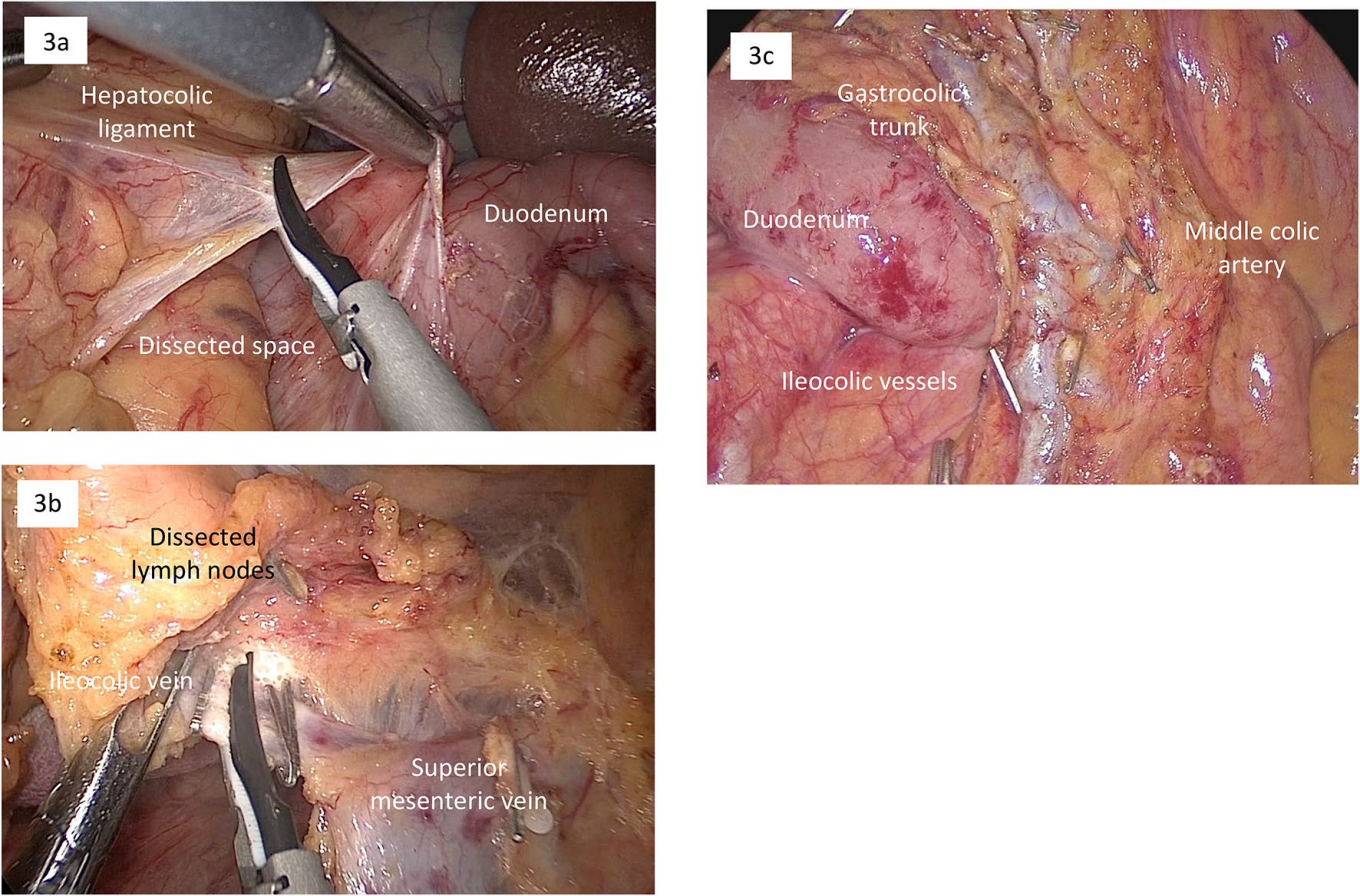
Surgical steps in the duodenum-first multidirectional approach. **a** Takedown of the hepatic flexure from the ventral side of the transverse mesocolon followed by fenestration of the dorsal dissection layer. **b**, **c** The central lymph nodes, including those along the dorsal side of superior mesenteric vein branches, are completely dissected (**b** ileocolic vessels, **c** right branch of the middle colic artery)

First, the line of demarcation between the mesentery of the small intestine and the retroperitoneum was exposed. Then, the assistant retracted the mesentery like a fan by grasping the mesentery above the horizontal portion of the duodenum. Mesentery dissection in DMA began by cutting the peritoneum along the root of the mesentery above the horizontal portion of the duodenum. In CMA, dissection began from the caudal side of the mesenteric root (Fig. 1b). Assistants maintained proper retraction during dissection to provide an optimal surgical field, grasping the mesentery at two points and moving in accordance with the primary surgeon’s actions. The dissection was developed above the ventral layer of the duodenum and pancreas and continued toward the cranial side in DMA (Fig. 2a). The anterior pancreatic fascia, which covers the duodenum, connects with Toldt’s fusion fascia, a meshwork of tissues parallels to the retroperitoneum (Fig. 2b), as well as the posterior pancreatic fascia. Therefore, the dissection layer above Gerota’s fascia and the retroperitoneum was easily maintained (Fig. 2c, d). During development of the dissection plane, the assistant used gauze to lightly lift the small bowel mesentery without causing injury and maintain a wide surgical field above the duodenum (Fig. 2a). As the dissection continued from the caudal root of the mesentery, the assistant’s hand moved to the mesentery of the terminal ileum to maintain appropriate tension. Since the ileocecal vessels may move from their original position after mobilization is completed, distorting their path, the lateral attachment of the cecum was left partially affixed. This maintains the ileocecal vessels in their original position before CVL. Next, the surface of the pancreatic head and the duodenum was exposed from the ventral side of the transverse mesocolon. After the accessory right colic vein (ARCV), also known as the superior right colic vein [17], was identified coursing into the gastrocolic trunk or the superior mesenteric vein (SMV), it was divided. Takedown from the hepatic flexure continued caudally, followed by fenestration of the dorsal dissection layer. Wide dorsal dissection above the duodenum helped early fenestration from the ventral side (Fig. 3a). Mobilization was then almost completed, except for a short part of the lateral attachment around the cecum. Finally, lymph node dissection with CVL was started in the same manner as for the conventional medial approach. After the assistant grasped the ileocecal vessels, the mesentery was cut on the caudal side of the vessels, which allowed easy fenestration into the dorsal dissection layer. Then, dissection of adipose tissue, including lymph nodes, on the ventral side of the SMV and superior mesenteric artery (SMA) was continued caudal-to-cranial to expose the branch origins. Next, the ileocolic vessels and right colic artery (if present) were cut at their root (Fig. 3b). For tumors located in the transverse colon, lymph node dissection around the middle colic artery (MCA) was usually required, and the right branch of the MCA was cut at its root (Fig. 3c). Complete mobilization of the right colon was then completed following dissection of the remaining lateral attachment around the cecum (supplementary video).

### Statistical analysis

Statistical analysis was performed using JMP software version 14.2.0 (SAS Institute, Cary, NC, USA). Patients were grouped according to the different surgical approaches and compared using univariate analysis. Categorical variables were compared using the *χ*^2^ test. Continuous variables were compared using the Wilcoxon rank-sum test. One-way analysis of variance was used to compare two or more continuous variables. *P* < 0.05 was considered significant.

## Results

### Patient characteristics

Patient age, sex, body mass index, clinical stage, surgical history, and surgical procedure did not significantly differ between multidirectional approach patients and conventional medial approach patients. There were 54 patients in the multidirectional group [29 females, median age 72 years (range 36–91 years)] and 66 in the medial group [42 females, median age 72 years (range 41–91 years)] A greater percentage of multidirectional approach patients underwent radical central lymph node dissection, although that did not reflect the number of harvested lymph nodes. Resected vessels did not significantly differ between the two groups (Table 1).

**Table 1:** Patient characteristics according to laparoscopic approach

		Approach		
		Multidirectional	Medial	*P* value
		*n* = 54	*n* = 66	
Age (years)	Median, (range)	72 (36—91)	72 (41—91)	0.96
Sex	Male	25 (46.3%)	24 (36.4%)	0.27
	Female	29 (53.7%)	42 (63.6%)	
BMI (kg/m^2^)	Mean, (range)	22.7 (14.8–36.8)	21.6 (14.9–30.6)	0.31
cStage	0	2 (3.7%)	2 (3.0%)	0.80
	I	15 (27.8%)	25 (37.9%)	
	II	14 (25.9%)	17 (25.8%)	
	III	17 (31.5%)	16 (24.2%)	
	IV	6 (11.1%)	6 (9.1%)	
Surgical history	Present	23 (42.6%)	21 (31.8%)	0.22
	Absent	31 (57.4%)	45 (68.2%)	
Lymph node dissection, including the area of the root of the main feeding vessels				
	Performed	44 (81.5%)	39 (59.1%)	0.007*
Resected vessels**	ICA	54 (100%)	66 (100%)	–
	RCA	10 (18.5%)	17 (25.8%)	0.34
	MCArt	17 (31.4%)	19 (28.8%)	0.75
	MCAlt	5 (9.3%)	3 (4.6%)	0.31

*BMI* body mass index, *ICA* ileocolic artery, *RCA* right colic artery, *MCArt* right branch of the middle colic artery, *MCAlt* left branch of the middle colic artery

*Indicates statistical significance (*p* < 0.05)

**Duplicate data

### Comparison of conventional medial and multidirectional approaches

Table 2 shows surgical outcomes and histological features according to surgical approach. Median operation time was significantly shorter in multidirectional approach patients than conventional medial approach patients (208 min vs. 271 min; *p* = 0.01). Blood loss did not differ. Intraoperative injury occurred in one conventional medial approach patient: the ARCV was inadvertently injured during dissection around the head of the pancreas and repaired with clipping and coagulation. One multidirectional approach patient was intraoperatively converted to laparotomy because of large tumor size. Incidence of postoperative complications did not significantly differ between the two groups. Average postoperative hospital stay in the multidirectional and conventional approach groups was not significantly different. Tumor size, incidence rates of tumor invasion and positive margins, and numbers of harvested and metastatic lymph nodes did not significantly differ between groups.

**Table 2:** Comparison of conventional medial and multidirectional laparoscopic right colectomy approaches

		Approach		
		Multidirectional	Medial	*P* value
		*n* = 54	*n* = 66	
Operation time (minutes)	Median (range)	208 (139–454)	271 (134–494)	0.01*
Blood loss, (ml)	Median (range)	29 (0–250)	40 (0–211)	0.18
Intraoperative injury		0 (0.0%)	1 (1.5%)	0.38
Conversion to open surgery		1 (2.1%)	0 (0.0%)	0.18
Postoperative complications**	All grades	8 (14.8%)	10 (15.2%)	0.96
	Surgical site infection	2 (3.7%)	4 (6.1%)	0.55
	Abdominal abscess	1 (1.9%)	1 (1.5%)	0.88
	Ileus	2 (3.7%)	2 (3.0%)	0.84
	Anastomotic leakage	0 (0%)	0 (0%)	–
	Others	3 (5.6%)	5 (7.6%)	0.66
Postoperative hospital stay (days)	Median (range)	11.8 (7–17)	10.5 (7–28)	0.07
Tumor size (mm)	Median (range)	38 (0.5–90)	40 (1–110)	0.80
Number of harvested lymph nodes	Median(range)	28 (8–55)	29 (8–79)	0.30
Resection margin positive		0 (0.0%)	0 (0.0%)	–
pT	T1/2	20 (37.0%)	23 (34.9%)	0.80
	T3/4	34 (63.0%)	43 (65.2%)	
Lymph node metastasis	Positive	14 (25.9%)	21 (31.8%)	0.27
pStage	0	3 (5.6%)	6 (9.1%)	0.91
	I	14 (25.9%)	15 (22.8%)	
	II	20 (37.0%)	23 (34.8%)	
	III	11 (20.4%)	16 (24.2%)	
	IV	6 (11.1%)	6 (9.1%)	

*Duplicate data

**Clavien–Dindo classification

### Comparison of CMA and DMA

Among multidirectional approach patients, operation time was significantly shorter in DMA patients than CMA patients (201 min vs. 269 min; *p* < 0.001). Operative time of the mobilization procedure was also significantly shorter in DMA patients (131 min vs. 181 min; *p* < 0.001). There was less blood loss in DMA patients, but the difference was not significant (20 mL vs. 40 mL; *p* = 0.08). The incidence of postoperative complications did not differ (Table 3). The average number of retraction adjustments was significantly lower in DMA patients (4.2 times vs. 6.4 times; *p* < 0.001).

**Table 3:** Comparison of duodenum-first and caudal-first multidirectional laparoscopic right colectomy approaches

		Multidirectional approach		
		DMA	CMA	*P* value
		*n* = 20	*n* = 34	
Operation time (minutes)	Median (range)	201 (139–289)	269 (191–454)	< 0.001*
Blood loss, (ml)	Median (range)	20 (0–250)	40 (0–247)	0.08
Intraoperative injury		0 (0.0%)	0 (0.0%)	–
Conversion to open surgery		0 (0.0%)	1 (2.9%)	0.42
Postoperative complications**	All	2 (10.0%)	6 (17.7%)	0.43
	Surgical site infection	1 (5.0%)	1 (2.9%)	0.70
	Abdominal abscess	1 (5.0%)	0 (0%)	0.16
	Ileus	0 (0%)	2 (5.9%)	0.27
	Anastomotic leakage	0 (0%)	0 (0%)	–
	Others	0 (0%)	3 (8.8%)	0.17
Postoperative hospital stay, (days)	Median (range)	11.8 (7–17)	11.9 (9–15)	0.86
Tumor size (mm)	Median (range)	37 (17–85)	38 (0.5–90)	0.92
Number of harvested lymph nodes	Median (range)	28 (12–55)	24 (8–52)	0.67
Resection margin positive		0 (0.0%)	0 (0.0%)	–
pT	T1/2	7 (35.0%)	13 (38.2%)	0.81
	T3/4	13 (65.0%)	21 (61.8%)	
Lymph node metastasis	Positive	5 (25.0%)	9 (26.5%)	0.91

*DMA* duodenum-first multidirectional approach, *CMA* caudal-first multidirectional approach

*Indicates statistical significance (*p* < 0.05)

**Duplicate data

### Comparison of conventional medial and multidirectional approaches in advanced tumors

In 77 patients with advanced T3/T4 tumors, DMA, CMA, and the conventional medial approach were performed in 13, 21, and 43 patients, respectively. Table 4 shows surgical outcomes and histological features according to surgical approach among patients with T3/T4 tumors. Operation time was significantly shorter in the DMA group (202 min) than the CMA (271 min) and conventional medial approach groups (295 min; *p* < 0.001). Operative time of the mobilization procedure was also significantly shorter in the DMA group (148 min) than the CMA (183 min) and conventional medial approach groups (180 min; *p* = 0.005). Blood loss, incidence of postoperative complications, and length of postoperative hospital stay did not significantly differ. R0 resection was achieved in all patients with T3/T4 tumors.

**Table 4:** Comparison of conventional medial and multidirectional laparoscopic right colectomy approaches in patients with advanced T3/T4 tumors

		Approach			
		DMA	CMA	Medial	*P* value
		*n* = 13	*n* = 21	*n* = 43	
Operation time, (minutes)	Median (range)	202 (139–280)	271 (216–443)	295 (225–494)	< 0.001*
Blood loss, (ml)	Median (range)	22 (0–75)	30 (0–247)	40 (0–155)	0.30
Intraoperative injury		0 (0.0%)	0 (0.0%)	1 (1.5%)	0.38
Conversion to open surgery		0 (0.0%)	1 (4.8%)	0 (0.0%)	0.27
Postoperative complications**	All	2 (20.0%)	4 (19.1%)	6 (14.0%)	0.82
Surgical site infection		1 (10.0%)	0 (0.0%)	3 (7.0%)	0.24
	Abdominal abscess	1 (10.0%)	0 (0%)	1 (2.3%)	0.30
	Ileus	0 (0.0%)	2 (9.5%)	2 (4.7%)	0.42
	Others	0 (0.0%)	2 (9.5%)	2 (4.7%)	0.42
Postoperative hospital stay (days)					
Median (range)		11.9 (8–17)	11.9 (8–17)	10.4 (7–27)	0.31
Tumor size (mm)	Median (range)	51 (17–85)	42 (15–90)	50 (6–110)	0.57
Number of harvested lymph nodes median (range)		25 (8–52)	28 (12–55)	30 (8–72)	0.46
Resection margin positive		0 (0.0%)	0 (0.0%)	0 (0.0%)	–
Lymph node metastasis	Positive	5 (38.5%)	8 (38.1%)	18 (41.9%)	0.95

*DMA* duodenum-first multidirectional approach, *CMA* caudal-first multidirectional approach

*Indicates statistical significance (*p* < 0.05)

**Duplicate data

## Discussion

On the basis of our results, the multidirectional approach in laparoscopic right colectomy is safe and associated with shorter operative time than the conventional medial approach. Among the 2 multidirectional approaches, DMA was associated with a much shorter operative time required for mobilization. We believe that standardizing the DMA can minimize the number of times required for changing the surgical field view. DMA provides a stable surgical plane during laparoscopic dorsal dissection, which allows easy recognition of the dissection layer and preserves retroperitoneal tissues and organs without resulting in intraoperative complications.

Although the conventional medial approach is simple, easy to understand, and widely accepted, considerable vascular variation and anatomical complexity around the pancreas can increase the technical difficulty of this approach [17–19]. Dissection and vessel ligation around the duodenum and pancreas through the deep and narrow peritoneal window can be arduous. The cranial-to-caudal approach can overcome this problem [20, 21]. This approach begins by identifying the pancreas and duodenum with exposure of the medial colic vessels and gastrocolic trunk of Henle from the ventral side of the transverse mesocolon. The resulting wide ventral view allows easy recognition and division of the ARCV and middle colic vein (MCV) at their origins and dissection of the mesentery along the SMV. However, potential complications include injury to SMA branches and incomplete clearance during CVL because forceps movement through the laparoscopic ports does not align with the direction of dissection. More recent approaches that start the dorsal dissection of the mesentery from the retroperitoneum (the retromesenteric or caudal-to-cranial approaches) are also feasible [6, 22, 23]. With these approaches, dissection proceeds from the root of the intestinal mesentery, then the broad view is explored during dorsal dissection. However, these approaches are still in development and have not been standardized. An optimal approach capable of widespread use has not yet been established.

During embryonic development, the small intestinal mesentery is attached to the duodenum and pancreas via the anterior pancreatic fascia and to Gerota’s fascia through Toldt’s fusion fascia [24, 25] (Fig. 1c). Lack of neurovascular bundles in these fasciae render them suitable for dissection in the retromesenteric approach [6, 22, 23]. Although the marginal and peripheral branches of the middle colic artery are accompanied by their draining veins, the central parts of these vessels run in different directions in the transverse mesocolon. The venous confluence around the pancreatic head is easily identified from the cranial side of the transverse mesocolon whereas the SMA confluence is easily recognized on the ventral side of the mesocolon [26]. Therefore, central veins can be safely divided using the cranial approach, and dissection along the SMA and SMV can be performed simply using the medial approach. Given the anatomical safety and limited movement of the laparoscopic forceps, the multidirectional approach utilizes the unique advantages of the three different approaches and is considered optimal for laparoscopic right colectomy [27]. However, the multidirectional approach requires at least three changes in the surgical field because of the need to repeatedly flip the colon, which can increase operative time. Although laparoscopic surgeons can choose multiple approaches in any combination for right colectomy, few studies have reported technical tips or a standardized procedure that clarifies proper surgical order. We have focused on duodenum-first mesenteric dissection, which can maintain the dissection layer above Toldt’s fusion fascia and avoid penetrating the fasciae, as the important first step in right colectomy. The wide and stable surgical field provided by the DMA permits easy recognition and preservation of the retroperitoneal organs with minimal change in assistant movements, which results in a shorter operative time compared with the conventional medial approach and retromesenteric dissection from the caudal side.

Three characteristics of our DMA procedure are critical: (1) appropriate countertraction to the assistant’s gentle retraction during cranial dissection; (2) early fenestration followed by wide dorsal dissection and (3) fixed traction to allow easy recognition of the ileocolic vessels as the first step of CVL. These key points are easily reproducible and should allow standardization and widespread acceptance of the DMA in laparoscopic right colectomy.

**Fig. 4:**
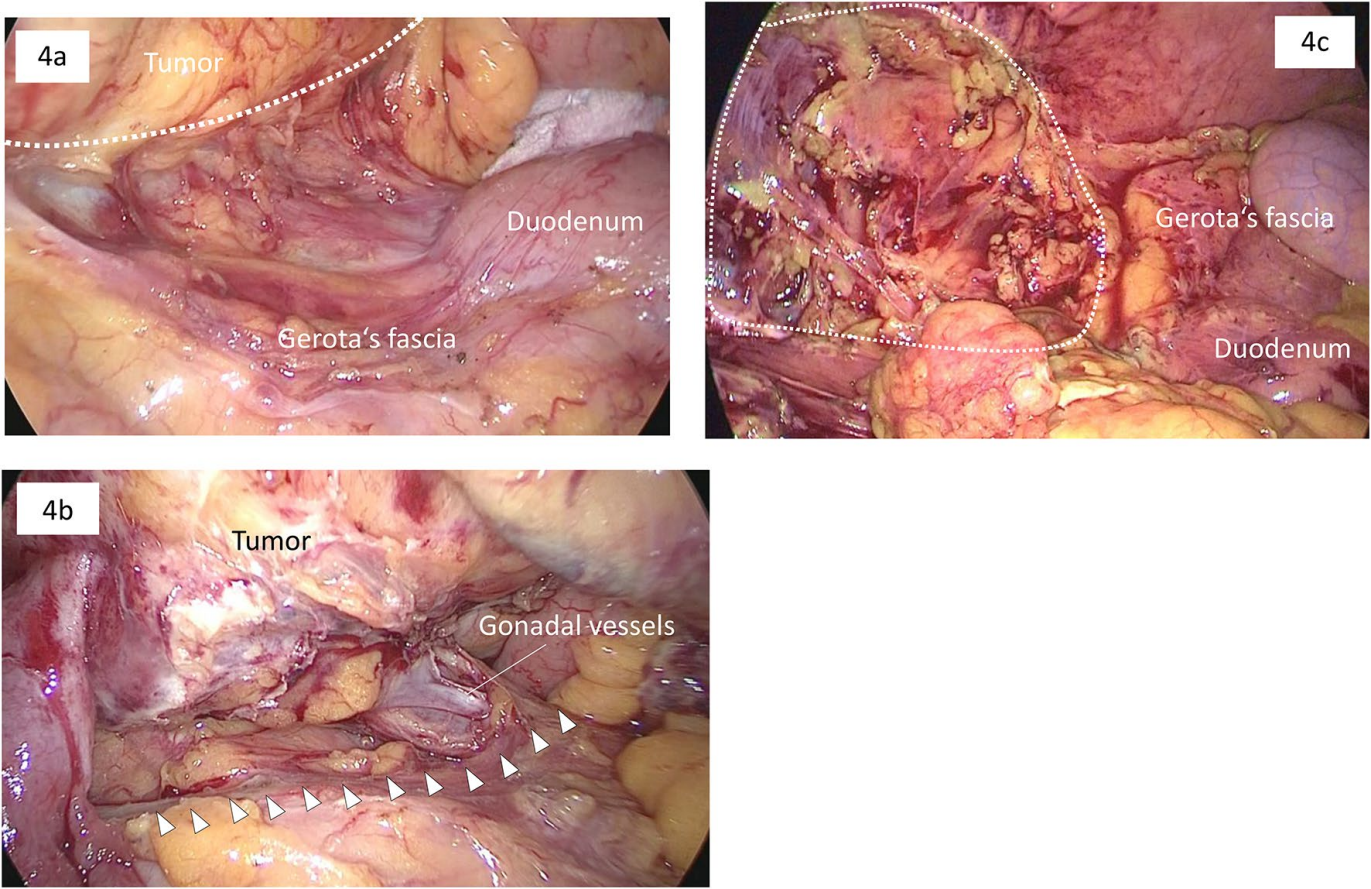
A patient with an advanced tumor in the ascending colon. **a** The dorsal resection margin (white dotted line) is easily recognized in the wide surgical view. **b** The tumor involving the right ovary and gonadal vessels is resected with the surrounding retroperitoneal tissue. **c** The white dotted line shows the space left after tumor resection

Complete oncological resection with harvesting of a sufficient number of lymph nodes was safely achieved in all multidirectional approach patients in our study. However, long-term outcome studies are required to confirm our results. Previous studies that evaluated patients with T3/T4 tumors have shown that laparoscopic surgery can adhere to surgical oncologic principles, is feasible and safe, and is associated with encouraging short-term outcomes compared with open surgery [28, 29]. However, to our knowledge, no study has examined laparoscopic right colectomy feasibility according to approach in these tumors. The wide view provided in the DMA helps to achieve adequate resection margins on the dorsal side (Fig. 4). We suggest that the DMA has potential for cure in patients with advanced tumors.

This study has several limitations. First, it was a retrospective single-center study with small sample size; firm conclusions cannot be drawn from our results. Second, we were unable to demonstrate that the DMA directly reduced the incidence of intraoperative injury to the surrounding organs compared with the conventional approach because this type of adverse event did not occur. CME is evaluated by pathological grading of the surgical resection plane [30]. Although no positive resection margins were detected and all resected specimens were evaluated to confirm complete macroscopic resection, the pathological reports did not contain pathological grading data. Future long-term prospective controlled outcome studies comparing the DMA and conventional approach are needed.

## Conclusions

The DMA in laparoscopic right colectomy is safe and feasible. This approach provides a stable surgical field, reduces operative time and is suitable for treating advanced tumors.

## Supplementary Information

Below is the link to the electronic supplementary material.

Supplementary file1 (MP4 401480 KB)

## Data Availability

The datasets generated during and/or analyzed during the current study are available from the corresponding author upon reasonable request.

## Declarations

### Informed conscent

Informed consent was obtained in the form of opt-out on the web-site.

## References

[1] West NP, Hohenberger W, Weber K, Perrakis A, Finan PJ, Quirke P (2010). Complete mesocolic excision with central vascular ligation produces an oncologically superior specimen compared with standard surgery for carcinoma of the colon. J Clin Oncol.

[2] Bae SU, Saklani AP, Lim DR (2014). Laparoscopic-assisted versus open complete mesocolic excision and central vascular ligation for right-sided colon cancer. Ann Surg Oncol.

[3] Siani LM, Lucchi A, Berti P, Garulli G (2017). Laparoscopic complete mesocolic excision with central vascular ligation in 600 right total mesocolectomies: safety, prognostic factors and oncologic outcome. Am J Surg.

[4] Shiroshita H, Inomata M, Bandoh T (2019). Endoscopic surgery in Japan: the 13th national survey (2014–2015) by the Japan Society for Endoscopic Surgery. Asian J Endosc Surg.

[5] Matsuda T, Iwasaki T, Mitsutsuji M (2015). Cranial-to-caudal approach for radical lymph node dissection along the surgical trunk in laparoscopic right hemicolectomy. Surg Endosc.

[6] Bae SU, Kim CN (2015). Laparoscopic complete mesocolic excision and central vascular ligation for right-sided colon cancer using the retroperitoneal approach. Dis Colon Rectum.

[7] Zhang Y, Sun DL, Chen XM (2017). The uncinate process first approach in laparoscopic pancreaticoduodenectomy: a single-institution experience. Surg Laparosc Endosc Percutan Tech.

[8] Feng B, Sun J, Ling TL (2012). Laparoscopic complete mesocolic excision (CME) with medial access for right-hemi colon cancer: feasibility and technical strategies. Surg Endosc.

[9] Hasegawa S, Kawamura J, Nagayama S, Nomura A, Kondo K, Sakai Y (2007). Medially approached radical lymph node dissection along the surgical trunk for advanced right-sided colon cancers. Surg Endosc.

[10] Mori S, Baba K, Yanagi M (2015). Laparoscopic complete mesocolic excision with radical lymph node dissection along the surgical trunk for right colon cancer. Surg Endosc.

[11] Ding J, Liao GQ, Xia Y (2013). Medial versus lateral approach in laparoscopic colorectal resection: a systematic review and meta-analysis. World J Surg.

[12] Xu P, Ren L, Zhu D (2015). Open right hemicolectomy: lateral to medial or medial to lateral approach?. PLoS ONE.

[13] Liang JT, Lai HS, Lee PH (2007). Laparoscopic medial-to-lateral approach for the curative resection of right-sided colon cancer. Ann Surg Oncol.

[14] Li F, Zhou X, Wang B (2017). Comparison between different approaches applied in laparoscopic right hemi-colectomy: a systematic review and network meta-analysis. Int J Surg.

[15] Y Hashiguchi K Muro Y Saito et al Japanese Society for Cancer of the C, Rectum (2020). Japanese Society for Cancer of the Colon and Rectum (JSCCR) guidelines 2019 for the treatment of colorectal cancer. Int J Clin Oncol.

[16] Clavien PA, Sanabria JR, Strasberg SM (1992). Proposed classification of complications of surgery with examples of utility in cholecystectomy. Surgery.

[17] Jin G, Tuo H, Sugiyama M (2006). Anatomic study of the superior right colic vein: its relevance to pancreatic and colonic surgery. Am J Surg.

[18] Lee SJ, Park SC, Kim MJ, Sohn DK, Oh JH (2016). Vascular anatomy in laparoscopic colectomy for right colon cancer. Dis Colon Rectum.

[19] Ueki T, Nagai S, Manabe T (2019). Vascular anatomy of the transverse mesocolon and bidirectional laparoscopic D3 lymph node dissection for patients with advanced transverse colon cancer. Surg Endosc.

[20] Matsuda T, Iwasaki T, Sumi Y (2017). Laparoscopic complete mesocolic excision for right-sided colon cancer using a cranial approach: anatomical and embryological consideration. Int J Colorectal Dis.

[21] Feng B, Ling TL, Lu AG (2014). Completely medial versus hybrid medial approach for laparoscopic complete mesocolic excision in right hemicolon cancer. Surg Endosc.

[22] Li H, He Y, Lin Z (2016). Laparoscopic caudal-to-cranial approach for radical lymph node dissection in right hemicolectomy. Langenbecks Arch Surg.

[23] Zou LN, Xiong WJ, Mo DL (2016). Laparoscopic radical extended right hemicolectomy using a caudal-to-cranial approach. Ann Surg Oncol.

[24] Culligan K, Walsh S, Dunne C (2014). The mesocolon: a histological and electron microscopic characterization of the mesenteric attachment of the colon prior to and after surgical mobilization. Ann Surg.

[25] Sadler TW (2011). Langman's medical embryology.

[26] Matsuda T, Sumi Y, Yamashita K (2017). Anatomy of the transverse mesocolon based on embryology for laparoscopic complete mesocolic excision of right-sided colon cancer. Ann Surg Oncol.

[27] Xie D, Yu C, Gao C, Osaiweran H, Hu J, Gong J (2017). An optimal approach for laparoscopic D3 lymphadenectomy plus complete mesocolic excision (D3+CME) for right-sided colon cancer. Ann Surg Oncol.

[28] Roscio F, Bertoglio C, De Luca A, Frattini P, Scandroglio I (2012). Totally laparoscopic versus laparoscopic assisted right colectomy for cancer. Int J Surg.

[29] Magistro C, Lernia SD, Ferrari G (2013). Totally laparoscopic versus laparoscopic-assisted right colectomy for colon cancer: is there any advantage in short-term outcomes? A prospective comparative assessment in our center. Surg Endosc.

[30] West NP, Morris EJ, Rotimi O, Cairns A, Finan PJ, Quirke P (2008). Pathology grading of colon cancer surgical resection and its association with survival: a retrospective observational study. Lancet Oncol.

